# Mechanistic insights into the survival curve of HeLa cells with a short shoulder and their S phase-specific sensitivity^†^

**DOI:** 10.1093/jrr/rrad097

**Published:** 2023-12-26

**Authors:** Kenshi Komatsu, Hiroshi Tauchi

**Affiliations:** Radiation Biology Center, Graduate School of Biostudies, Kyoto University, Yoshida-konoecho, Sakyo, Kyoto-shi, Kyoto 606-8501, Japan; Department of Biological Sciences, Faculty of Science, Ibaraki University, Bunkyo 2-1-1, Mito, Ibaraki 310-8512, Japan

**Keywords:** radio-suppression of homologous recombination repair, HeLa cells, RIF1, short shoulder, intra-S checkpoint, cell cycle-dependent radiosensitivity

## Abstract

HeLa cells are a cell line with two unique cellular features: a short-shouldered survival curve and two peaks of radioresistance during the cell cycle phase, while their underlying mechanisms remain unclear. We herein proposed that these radiobiological features are due to a common mechanism by which radiation suppresses homologous recombination repair (HRR) in a dose-dependent manner. This radio-suppression of HRR is mediated by an intra-S checkpoint and reduces survivals of cells in S phase, especially early S phase, resulting in both short shoulder and radioresistance with two peaks in the cell cycle. This new explanation may not be limited to HeLa cells since a similar close association of these features is also observed in other type of cells.

HeLa cells, widely used in the field of radiobiology since their first application to dose-survival curves, are characterized by two unique radiobiological properties [1, 2]. The dose-survival curves for both human and mammalian cells have an initial shoulder followed by a steep decrease with increasing doses, and the shoulder is algebraically expressed by Dq, the quasi-threshold dose. HeLa cells have an extremely short shoulder, with Dq = 0.75 Gy, in contrast to 2.38 Gy for hamster V79 cells [1, 3]. Another feature of HeLa cells is the shape of radioresistance that appears with the progression of the cell cycle phase, which significantly differs from that of hamster V79 cells: hamster V79 cells have one radioresistance peak in the late S phase, while HeLa cells have two peaks in the G1 and late S phases (Fig. 1A) [2]. The difference in the age response between the HeLa cells and hamster V79 cells has been explained by the length of the G1 phase. That is, the pattern in HeLa cells is indistinguishable in hamster V79 cells because G1 is too short [2]. However, a long period for G1 phase is not essential for the appearance of two peaks of radioresistance since the murine lymphoma L5178Y cells have a short G1 phase (~1 h, comparable to hamster V79 cells) and exhibit the same age response as HeLa cells [4]. Therefore, an alternative explanation for the differences between HeLa cells and hamster V79 cells seems necessary. Additionally, L5178Y cells are characterized by a short shoulder, whereas a correlation of the opposite features of broad shoulder with single-peak radioresistance, as in hamster V79 cells, has also been reported in other cell lines, such as chicken DT40 cells [5, 6]. These findings suggest that the mechanism determining the length of the shoulder in the survival curve is related to the shape of radioresistance in the cell cycle phase. Recent studies at the molecular level indicated that these properties of HeLa cells were likely due to a common mechanism by which the homologous recombination repair (HRR) is suppressed after an exposure to radiation.

**Fig. 1:**
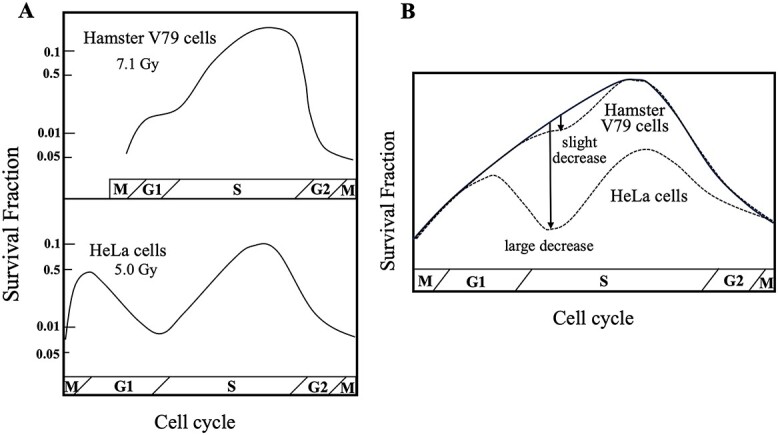
Differential shape of radioresistance associated with decreased cell survival in early S phase. (**A**) Hamster V79 cells have a single radioresistance peak in late S phase, while HeLa cells have two peaks in both G1 and late S phases, modified from [2]. (**B**) A slight decrease in the survival in early S phase forms a single peak of radioresistance, as observed in hamster V79 cells, whereas in HeLa cells, a large decrease resulted in a curve with two peaks of radioresistance.

Saito *et al*. quantitatively measured the activity of HRR in HeLa cells after an exposure to radiation by using an HRR reporter gene, which exhibited the radio-suppression of HRR [7]. Although HRR was intact in HeLa cells and rejoined I-SceI-induced DSBs in the absence of radiation, radiation significantly suppressed HRR in a dose-dependent manner, with decreases to 70 and 30% after the delivery of 1 and 3 Gy, respectively. Similar radio-suppression was observed in radiation-induced DSBs when HRR was measured by immunostaining for RAD51, a surrogate marker for HRR. Importantly, these RAD51 foci exclusively formed during the S-G2 phase, indicating that the radio-suppression of HRR occurred in the S or G2 phase. HRR was subsequently measured in cells depleted of Rap1-interacting factor 1 (RIF1) because RIF1 accumulates at DSB sites via the ATM-dependent phosphorylation of 53BP1 and inhibits HRR by protecting the DNA ends through the so-called 53BP1-RIF1-Shieldin pathway [8]. As expected, the depletion of RIF1 restored the suppression of HRR after irradiation, as measured by RAD51 staining, suggesting that it exerted its effects in S-G2 phase. Another well-known role of RIF1 is to coordinate the timing of replication during S phase in which RIF1 forms a complex with phosphatase 1 and then dephosphorylates MCM2 replication helicase to maintain it in an inactive state [9]. An immunochemical analysis of the gaps between the nascent DNA sites before and after irradiation revealed that the initiation of replication was transiently inhibited by irradiation (the so-called intra-S checkpoint) in wild-type cells, but not in RIF1-depleted cells [7, 10]. To investigate whether RIF1 functions in both the intra-S checkpoint and radio-suppression of HRR through a common pathway, the endogenous MCM2 protein, which is upstream of the intra-S checkpoint, was degraded using the auxin-inducible degron system, and wild-type MCM2 or a phospho-dead (S40A, S53A and S108A) MCM2 mutant was then reconstituted in HeLa cells [7]. The findings obtained showed that the introduction of the MCM2 mutant impaired both the intra-S checkpoint and HRR pathway, indicating that HRR was suppressed in HeLa cells by the intra-S checkpoint, i.e. its suppression occurred during S phase.

Since HRR and NHEJ are two major DNA repair processes that rejoin DSBs, the radio-suppression of HRR should mainly reduce the surviving fraction of cells in the early S phase, when the intra-S checkpoint is mostly activated [11]. A careful examination of the shapes of cell survival curves in each cell cycle phase in HeLa cells and hamster V79 cells revealed that both cell lines showed different degrees of decreases in cell survival in early S phase (Fig. 1B); a slight decrease in early S phase formed a single peak of radioresistance in hamster V79 cells, whereas a large decrease resulted in a curve with two peaks of radioresistance in HeLa cells. Consistent with this, inhibition of DNA synthesis after low-dose radiation is clearly different between the HeLa cell and hamster V79 cells: less radio-sensitive inhibition of DNA synthesis in hamster V79 cells (weak intra-S checkpoint) and marked radio-sensitive inhibition of DNA synthesis in HeLa cells (significant intra-S checkpoint) [12]. Thus, radio-suppression of HRR mediated by the intra-S checkpoint fully explains the cell survival significantly decreasing in the early S phase and resulting a two-peak shape of radioresistance in the cell cycle phase, as observed in HeLa.

Since S phase cells contribute to the formation of a dose-survival curve with a broad shoulder, their reduction of the survivals should shorten the shoulders [13]. This is also supported by studies of DNA repair showing that the suppression of HRR attenuates cell survivals, leading to shoulder shortening [14]. Therefore, HRR inactivation and subsequent S phase-specific radiosensitivity may account for the close relationship between the shorter shoulder in the survival curve and the two-peaked shape of radioresistance with the cell cycle phase, as observed in HeLa cells. However, it remains elusive why RIF1-mediated radiosensitization is not observed in hamster V79 cells. One possible explanation is that RIF1 function is antagonized by BRCA1 and that BRCA1 is induced at different expression levels in cells, particularly between tumor cells [7, 15, 16]. Although the expression ratio of RIF1 and BRCA1 might affect the radiosensitivity of the intra-S checkpoint, we cannot exclude the possibility of other proteins in the DNA damage response.

In conclusion, the radio suppression of HRR may be a mechanism for the marked reduction in cell survival during S phase, resulting in both the formation of a short shouldered survival curve and the two-peaked shape of radioresistance in the cell cycle. This concept must be generalized by further studies on the dose-dependent suppression of HRR in other cell lines and on the expression levels of related proteins, such as RIF1 and its antagonist.

## CONFLICT OF INTEREST

The authors declare that they have no conflict of interest.

## FUNDING

This work was supported by JSPS KAKENHI (grant number JP22K19847) to K.K.
